# Two new species of the Balkan genus *Paladilhiopsis* Pavlović, 1913 (Caenogastropoda, Moitessieriidae)

**DOI:** 10.3897/zookeys.1046.64489

**Published:** 2021-06-21

**Authors:** Sebastian Hofman, Jozef Grego, Aleksandra Rysiewska, Artur Osikowski, Andrzej Falniowski

**Affiliations:** 1 Department of Comparative Anatomy, Institute of Zoology and Biomedical Research, Jagiellonian University, ul. Gronostajowa 9, 30-387, Kraków, Poland; 2 Horná Mičiná 219, SK-97401, Banská Bystrica, Slovakia; 3 Department of Malacology, Institute of Zoology and Biomedical Research, Jagiellonian University, ul. Gronostajowa 9, 30-387, Kraków, Poland; 4 Department of Animal Reproduction, Anatomy and Genomics, University of Agriculture in Krakow, al. Mickiewicza 24/28, 30-059, Kraków, Poland

**Keywords:** Anatomy, COI, H3, molecular systematic, mOTU, radula, shell, stygobiont

## Abstract

The Balkan Peninsula is inhabited by the worldwide most diverse subterranean gastropod fauna. This fauna is still poorly studied, since its habitats are not easily accessible, and its sampled populations are mostly not rich in specimens’ numbers. Often only empty shells are known, but the shell is hardly useful, not only in phylogeny reconstruction, but even in species determination. The exclusively obligatory subterranean family Moitessieriidae is especially poorly studied. Representatives of the genus *Paladilhiopsis* Pavlović, 1913 (Moitessieriidae) collected at three localities, distributed in Croatia and Bosnia & Herzegovina, were studied. The pigmentation of their shells and soft parts, as well as the female and male reproductive organs in one taxon, are presented. The partial sequences of the molecular markers mitochondrial cytochrome oxidase subunit I (COI) and nuclear histone 3 (H3) were used to infer their systematic status and phylogenetic relationships. Two species new to science are described. For one of them, also studied anatomically, 15 specimens were sequenced for COI, and all show the same haplotype.

## Introduction

Gastropods are an important component of the subterranean fauna (Culver 2012), but are still poorly studied (e.g., Culver and Pipan 2009, 2014). Of the approximately 20,000 worldwide species of subterranean animals (Culver and Pipan 2009), there are more than 350 described species of stygobiont (obligate subterranean aquatic) gastropods, 97% of them belong to the Hydrobiidae sensu lato (Bernasconi and Riedel 1994; Culver 2012), representing several families belonging to the Truncatelloidea (Criscione and Ponder 2013). The most diverse subterranean gastropod fauna worldwide inhabits the Balkan Peninsula, mainly the Dinaric karst. Sket et al. (2004) reported 169 obligate stygobiont gastropod species inhabiting this territory. As stressed by Falniowski (2018), variable shells as the only known structures, coupled with the widespread, dogmatic belief in geographic isolation and unavoidable, immediate speciation, resulted in descriptions of new species (nearly) in each cave or other subterranean habitat. Low densities of subterranean populations, coupled with not easily penetrable habitats, resulted in numerous nominal species known only as empty shells, washed out into springs at times of high flow, especially during spring (Haase 1995; Richling et al. 2016). Even the soft parts, if accessible, were not usually informative enough to resolve taxonomic questions, since the animals are very tiny and miniaturisation has resulted in simplification of their anatomy (e.g., Culver 2012; Falniowski 2018). Molecular data are helpful, but there are still only few studies applying them (e.g., Grego et al. 2019; Hofman et al. 2019).

All the restrictions of our knowledge outlined above are even more severe in the case of the family Moitessieriidae Bourguignat, 1863, whose monophyly has recently been proved (Falniowski et al. 2019). Its representatives are minute gastropods, all of them obligatory subterranean, exclusively inhabiting subterranean waters, including thermal ones (Sket and Velkovrh 1981). High variability in shell morphology and the lack of diagnostic features in the morphology of the simplified soft parts, coupled with anticipated high levels of endemism has resulted in a long list of nominal moitessieriid species (see e.g., Glöer 2002 for *Bythiospeum* Bourguignat, 1882). The anatomy of the family is still poorly known and provided only for a few taxa. Detailed anatomy of *Bythiospeum* Bourguignat, 1882 was described and illustrated by Haase (1995) and Girardi and Rosello (2001). Some anatomical data on the Moitessieriidae were contributed also by Bole (1961, 1970), Giusti and Pezzoli (1980) (anatomy of *Iglica* Wagner, 1927 and *Paladilhiopsis* Pavlović, 1913), Radoman (1983), Bernasconi (1990, 1994), Boeters and Gittenberger (1990), Bodon and Giusti (1991), Boeters (1998), Szarowska (2006), Niero and Pezzoli (2016), and Hofman et al. (2018).

The genus *Paladilhiopsis* Pavlović, 1913 (type species *Paladilhia robiciana* Clessin, 1882), inhabiting the Balkans (including Hungary) was considered as a subgenus of *Bythiospeum* by Slapnik (1995). Boeters (1998) synonymised *Paladilhiopsis* with *Bythiospeum*, based on the similarity of the general organisation of the female reproductive organs, i.e., a large bursa copulatrix situated at the proximal part of the albumen gland, which is markedly shortened. In our partial revision of the Balkan Moitessieriidae (Hofman et al. 2018) we confirmed the anatomy of the female reproductive organs of *Paladilhiopsis*, but our molecular data definitely proved rather distant phylogenetic relationships between *Paladilhiopsis* and *Bythiospeum*, unequivocally classifying this morphological similarity (of very simple structures) as a homoplasy, certainly not a synapomorphy. Continued field collection, applying also the Bou-Rouch technique for collection of interstitial gastropods, resulted in some new *Paladilhiopsis*, which were checked for molecular markers. Their phylogenetic position, applying the shell, soft parts morphology (if the material was available) and molecular distinctness and relationships are the subject of the present paper.

## Materials and methods

**Figure 1.**
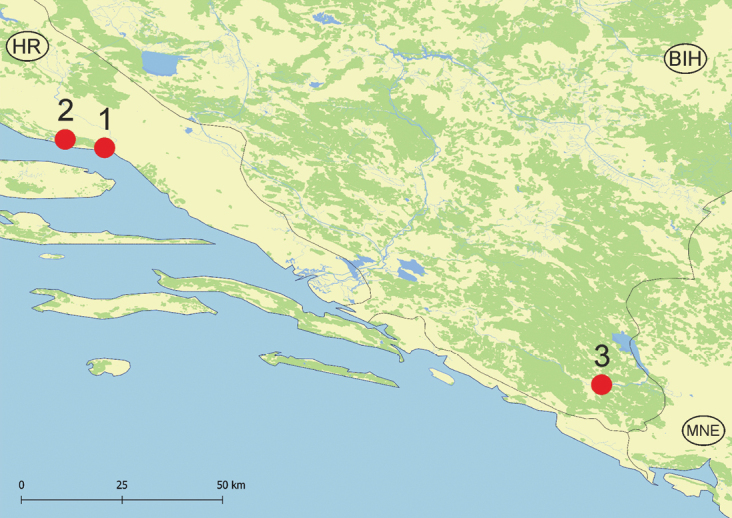
Localities map.

**Figure 2.**
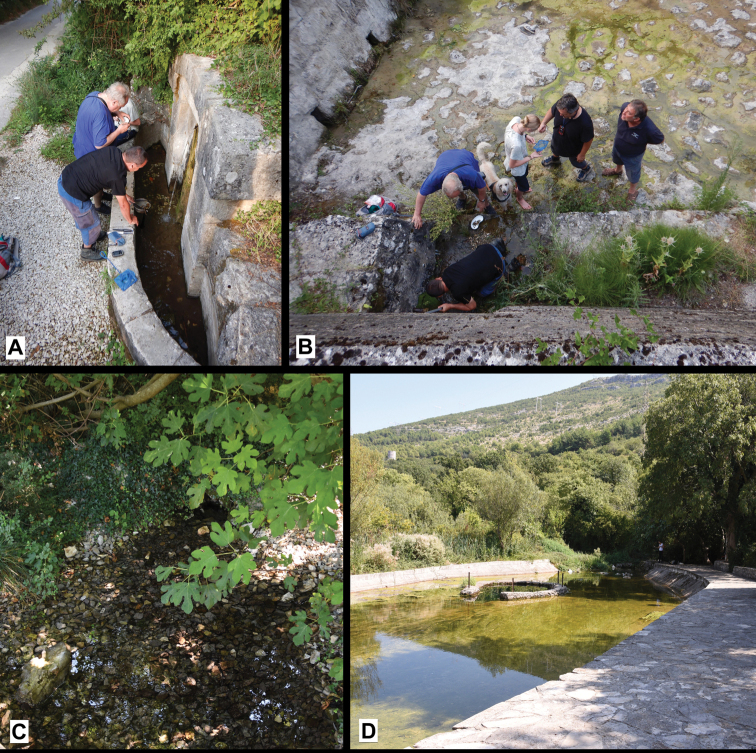
Some of the sampled localities **A** Studena spring (loc. 1) **B** Spring Zvezda (loc. 2) **C, D** Vrelo „Lušac” (Gučina) (loc. 3) (photographs **A, B** M. Olšavský **C, D** A. Osikowski).

The snails were collected at three localities (Table 1), distributed in Bosnia and Herzegovina and Croatia (Figs 1, 2). They were either collected by hand and sieve in springs, or with a pump applying Bou-Rouch technique (Bou and Rouch 1967), to sample interstitial fauna below the bottom of streams, at the depth of ca. 50 cm. The tube was inserted in the bottom five times, and 20 litres were pumped each time. Samples were sieved through 500 μm sieve and fixed in 80% analytically pure ethanol, replaced twice, and later sorted. Next, the snails were put in fresh 80% analytically pure ethanol and kept at -20 °C temperature in a refrigerator.

**Table 1.** Sample localities.

Id #	Site name	Coordinates
1	Studena spring, left bank of Cetina River, Slime, Croatia, locality G25	43°25'43"N, 16°51'59"E
2	Spring Zvezda, above left bank of Cetina, Croatia, locality 26	43°26'13"N, 16°44'26"E
3	Vrelo „Lušac” (Gučina), BiH, locality 19-10a	42°42'04"N, 18°21'27"E

The shells were photographed with a Canon EOS 50D digital camera, under a Nikon SMZ18 microscope with a dark field. The dissections were done under a Nikon SMZ18 microscope with dark field, equipped with Nikon DS-5 digital camera, whose captured images were used to draw anatomical structures with a graphic tablet. The penes were photographed under Motic microscope with dark field. The shells were cleaned with an ultrasonic cleaner, the radulae were extracted with Clorox, applying the techniques described by Falniowski (1990), and examined and photographed using a HITACHI S-4700 scanning electron microscope. Morphometric parameters of the shell (following the scheme of Falniowski et al. 2007) were measured by one person using a Nikon DS-5 digital camera and ImageJ image analysis software (Rueden et al. 2017).

Snails for molecular analysis were fixed in 80% ethanol, changed twice, and later stored in 96% ethanol. DNA was extracted from whole specimens; tissues were hydrated in TE buffer (3 × 10 min); then total genomic DNA was extracted with the SHERLOCK extraction kit (A&A Biotechnology), and the final product was dissolved in 20 μl of tris-EDTA (TE) buffer. The extracted DNA was stored at –80 °C at the Department of Malacology, Institute of Zoology and Biomedical Research, Jagiellonian University in Kraków (Poland).

Mitochondrial cytochrome oxidase subunit I (COI), and nuclear histone 3 (H3) loci were sequenced. Details of PCR conditions, primers used, and sequencing were given in Szarowska et al. (2016). Sequences were initially aligned in the MUSCLE (Edgar 2004) program in MEGA 7 (Kumar et al. 2016) and then checked in BIOEDIT 7.1.3.0 (Hall 1999). Uncorrected p-distances were calculated in MEGA 7. In the phylogenetic analysis additional sequences from GenBank were used as reference (Table 2). The estimation of the proportion of invariant sites and the saturation test (Xia 2000; Xia et al. 2003) were performed using DAMBE (Xia 2013). The data were analysed using approaches based on Bayesian Inference (BI) and Maximum Likelihood (ML). We applied the GTR model whose parameters were estimated by RAxML (Stamatakis 2014). The General Time Reversible (GTR) model is the most complicated one, including all the simpler cases assumed by the other models. We agree with the arguments of Stamatakis that the simpler models are only computationally less expensive, which is of diminishing importance with modern computers. At the same time, as pointed out already by Nei and Kumar (2000), the criteria like AIC often collapse in choosing the proper model of DNA evolution. With several data sets, MODELTEST and MEGA totally collapsed in attempting to find the proper model (pers. obs. AF).

**Table 2.** Taxa used for phylogenetic analyses with their GenBank accession numbers and references.

Species	COI/H3 GB numbers	References
*Bythiospeum acicula* (Hartmann, 1821)	KU341350/MK609534	Richling et al. 2016/Falniowski et al. 2019
*Bythiospeum alzense* Boeters, 2001	KU341354/–	Richling et al. 2016
*Ecrobia maritima* (Milaschewitsch, 1916)	KX355835/MG551322	Osikowski et al. 2016/Grego et al. 2017
Iglica cf. gracilis (Clessin, 1882)	MH720985–MH720986/ MH721002–MH721003	Hofman et al. 2018
*Iglica hellenica* Falniowski & Sarbu, 2015	KT825581/MH721007	Falniowski and Sarbu 2015/ Hofman et al. 2018
*Lanzaiopsis savinica* Bole, 1989	MN272428–MN272429/MN272430–MN272431	Prevorčnik et al. 2019
Moitessieria cf. puteana Coutagne, 1883	AF367635/MH721012	Wilke et al. 2001/ Hofman et al. 2018
Paladilhiopsis cf. absoloni (A. J. Wagner, 1914)	–/MH721021	Hofman et al. 2018
*Paladilhiopsis blihensis* (Glöer & Grego, 2015)	–/MH721015	Hofman et al. 2018
*Paladilhiopsis bosniaca* (Clessin, 1910)	–/MH721020	Hofman et al. 2018
*Paladilhiopsis bosnica* Bole, 1970	–/MH721021	Hofman et al. 2018
*Paladilhiopsis grobbeni* Kuščer, 1928	MH720991/MH721014	Hofman et al. 2018
*Paladilhiopsis turrita* (Kuščer, 1933)	MH720992/MH721015	Hofman et al. 2018
*Paladilhiopsis gittenbergeri* (A. Reischutz & P. L. Reischutz, 2008)	MH720993/MH721025	Hofman et al. 2018
*Paladilhiopsis matejkoi* Glöer & Grego, 2019	MK632245/MK632246	Grego et al. 2019
*Paladilhiopsis maroskoi* (Glöer & Grego, 2015)	–/MH721017	Hofman et al. 2018
*Paladilhiopsis montenegrinus*	MW452318–MW452319/MW452604–MW452605	Rysiewska et al. unpub.
*Pseudamnicola pieperi* (Westerlund, 1886)	KT710668/KT710740	Szarowska et al. 2016

The Bayesian analyses were run using MrBayes v. 3.2.3 (Ronquist et al. 2012) with defaults of most priors. Two simultaneous analyses were performed, each with 10,000,000 generations, with one cold chain and three heated chains, starting from random trees and sampling the trees every 1,000 generations. The first 25% of the trees were discarded as burn-in. The analyses were summarised as a 50% majority-rule tree. Convergence was checked in Tracer v. 1.5 (Rambaut and Drummond 2009). The Maximum Likelihood analysis was conducted in RAxML v. 8.2.12 (Stamatakis 2014) using the ‘RAxML-HPC v.8 on XSEDE (8.2.12) tool via the CIPRES Science Gateway (Miller et al. 2010). Two species delimitation methods were performed: Poisson Tree Processes (PTP) (Zhang et al. 2013) and Automatic Barcode Gap Discovery (ABGD) (Puillandre et al. 2011). The PTP approach was run using the web server https://species.h-its.org/ptp/, with 100 000 MCMC generations, 100 thinning and 0.1 burn-in. We used RAxML output phylogenetic tree. The ABGD approach using the web server (https://bioinfo.mnhn.fr/abi/public/abgd/abgdweb.html) and the default parameters.

## Results

We obtained 17 new sequences of COI (457 bp, GenBank accession numbers MW741724–MW741740) and nine of H3 (310 bp, GenBank accession numbers MW776417–MW776425). The tests by Xia et al. (2003) revealed no saturation. In all analyses, the topologies of the resulting phylograms were identical in both the Maximum Likelihood (ML) and Bayesian Inference (BI).

**Figure 3.**
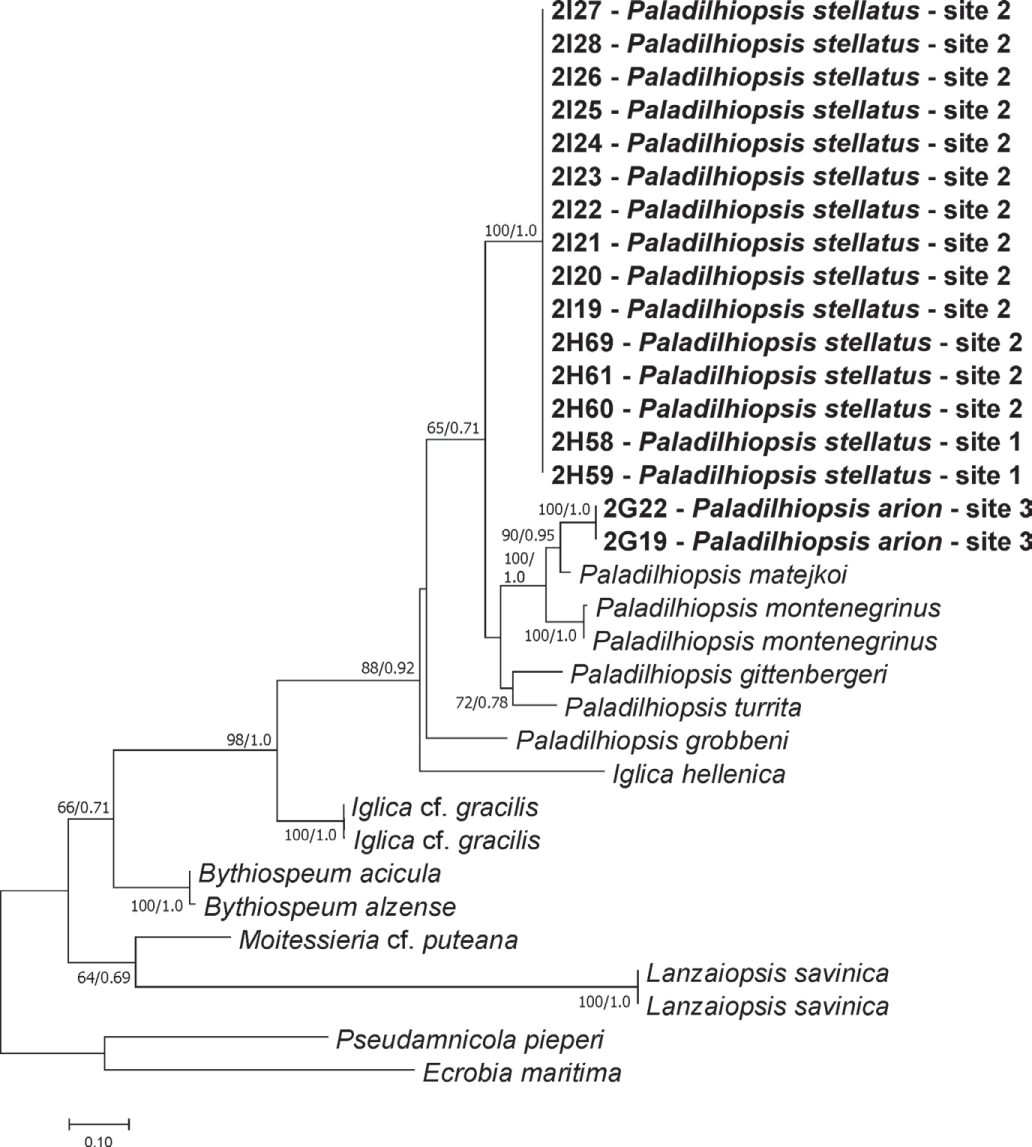
The maximum-likelihood phylogram for the COI gene. Bootstrap supports given if ≥ 60%.

**Figure 4.**
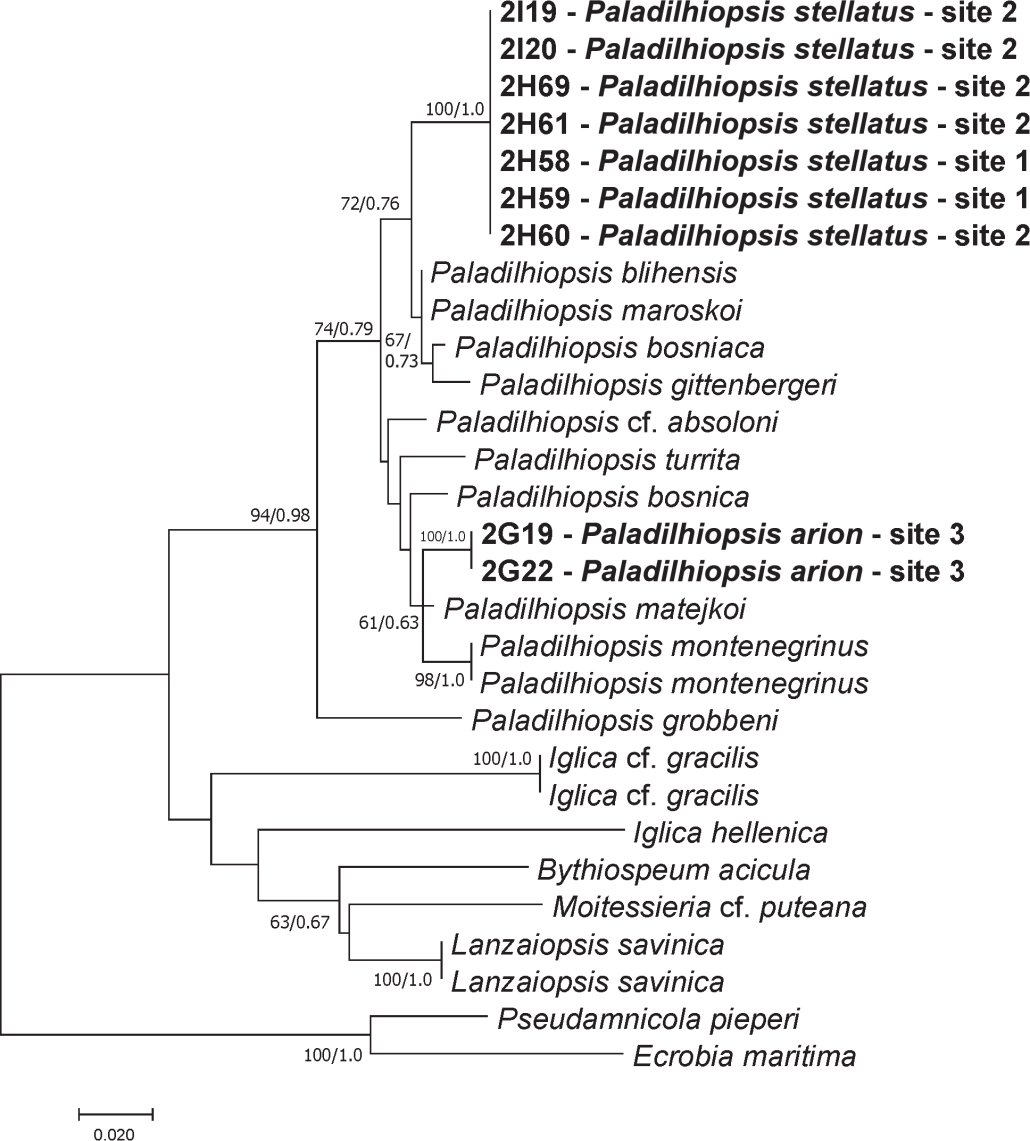
The maximum-likelihood phylogram for the H3 gene. Bootstrap supports given if ≥ 60%.

**Figure 5.**
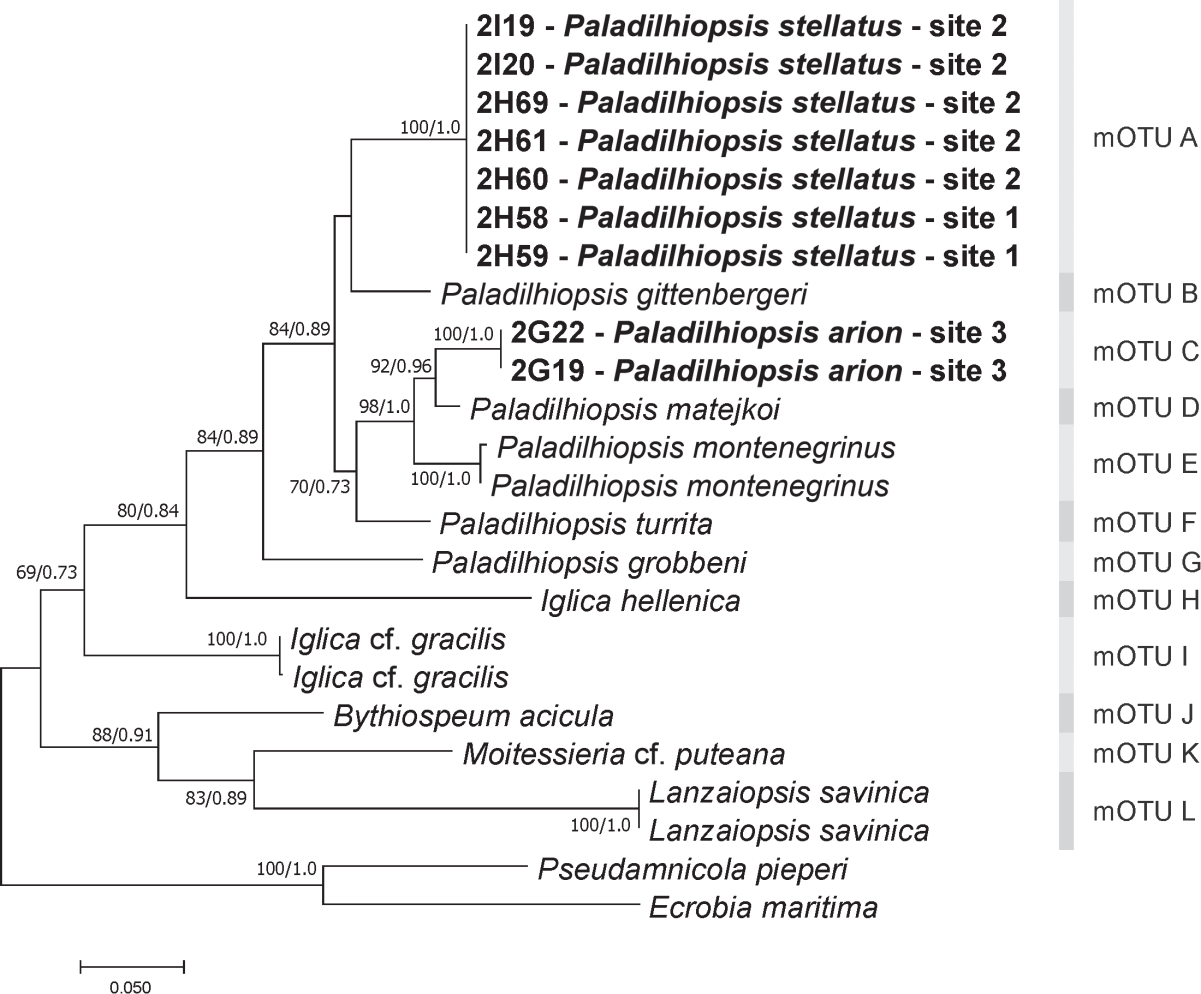
Maximum likelihood tree computed for concatenated partial sequences of COI and H3 sequences. Bootstrap supports given if ≥ 60%.

All newly sequenced specimens belonged to the Moitessieriidae at the COI (Fig. 3) as well as H3 (Fig. 4) trees. In the maximum likelihood tree computed for nine new concatenated sequences of the both studied loci, together with all the Balkan species of the Moitessieriidae whose COI and H3 sequences were available (Fig. 5), PTP and ABGD methods inferred twelve mOTUs (A-L), two of them (A, C) new, most probably of the species rank. The p-distances between mOTUs (Table 3) ranged from 0.059 to 0.298 for COI and from 0.016 to 0.142 for H3. All the new taxa belong to the genus *Paladilhiopsis*, as defined by Hofman et al. (2018). The levels of divergence for these new taxa were comparable to other *Paladilhiopsis* taxa of species rank (Table 3). At locality 2, as many as 13 specimens were sequenced for the cytochrome oxidase subunit I (COI), and no polymorphism was found in this variable locus; thus, only seven specimens were sequenced also for COI and the much more conservative histon 3 (H3) locus, showing no infrapopulation polymorphism.

**Table 3.** P-distances between main COI mOTUs of *Paladilhiopsis*. For details, see Fig. 5.

	A	B	C	D	E	F	G	H	I	J	K	L
**A**		**0.035**	**0.045**	**0.035**	**0.042**	**0.042**	**0.071**	**0.135**	**0.126**	**0.113**	**0.100**	**0.110**
**B**	**0.113**		**0.035**	**0.026**	**0.035**	**0.039**	**0.071**	**0.132**	**0.126**	**0.123**	**0.103**	**0.113**
**C**	**0.145**	**0.140**		**0.016**	**0.023**	**0.035**	**0.068**	**0.132**	**0.110**	**0.019**	**0.106**	**0.110**
**D**	**0.124**	**0.116**	**0.059**		**0.016**	**0.026**	**0.061**	**0.129**	**0.113**	**0.116**	**0.103**	**0.106**
**E**	**0.132**	**0.140**	**0.091**	**0.081**		**0.029**	**0.061**	**0.129**	**0.116**	**0.113**	**0.110**	**0.110**
**F**	**0.116**	**0.094**	**0.118**	**0.105**	**0.118**		**0.061**	**0.142**	**0.116**	**0.113**	**0.106**	**0.123**
**G**	**0.137**	**0.134**	**0.164**	**0.134**	**0.181**	**0.137**		**0.126**	**0.113**	**0.119**	**0.123**	**0.116**
**H**	**0.167**	**0.177**	**0.177**	**0.164**	**0.194**	**0.164**	**0.156**		**0.135**	**0.126**	**0.116**	**0.100**
**I**	**0.156**	**0.173**	**0.196**	**0.172**	**0.183**	**0.157**	**0.176**	**0.198**		**0.103**	**0.119**	**0.110**
**J**	**0.220**	**0.226**	**0.204**	**0.194**	**0.192**	**0.202**	**0.204**	**0.215**	**0.185**		**0.087**	**0.061**
**K**	**0.231**	**0.242**	**0.199**	**0.212**	**0.212**	**0.215**	**0.247**	**0.253**	**0.203**	**0.185**		**0.065**
**L**	**0.298**	**0.282**	**0.245**	**0.250**	**0.237**	**0.263**	**0.288**	**0.277**	**0.273**	**0.255**	**0.226**	

### Systematic part

#### Family Moitessieriidae Bourguignat, 1863

**Genus *Paladilhiopsis* Pavlović, 1913**

##### Paladilhiopsis stellatus

Taxon classificationAnimaliaLittorinimorphaMoitessieriidae

Grego & Hofman sp. nov.

3352875A-818B-5647-B707-AA71C8B010DB

http://zoobank.org/01ff1956-336a-4942-b634-1a5d036b4cdd

**Figure 6.**
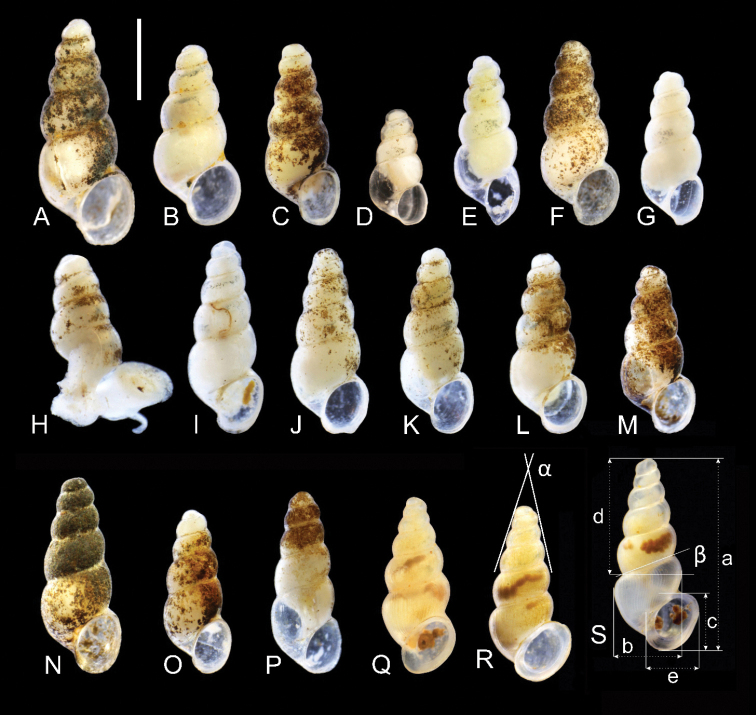
Shells of *Paladilhiopsis***A–P***P. stellatus***A, B** locality 1, Studena spring (2H58, 2H59) **C–P** locality 2, Zvezda spring (holotype, 2H69, 2H60-2H61, 2I19-2I28, respectively) **Q–S***P. arion*, locality 3, Vrelo „Lušac” (Gučina), BiH (holotype, 2G19, 2G22).

**Figure 7.**
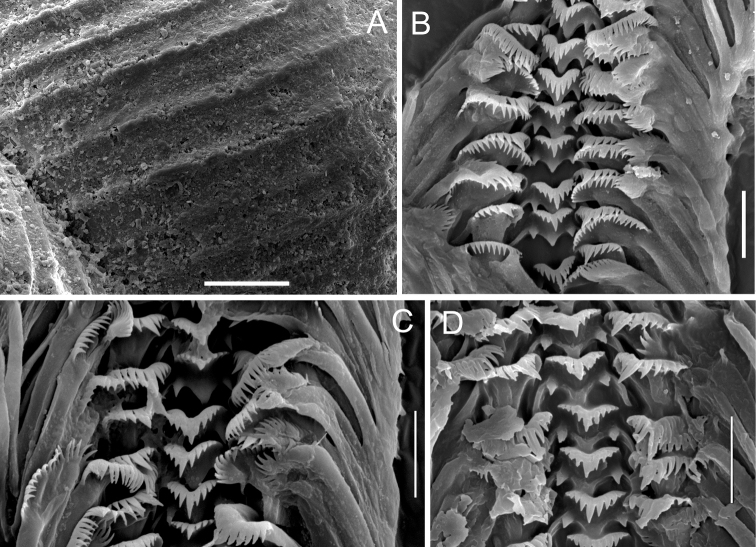
**A** teleoconch sculpture of *Paladilhiopsis arion***B–D** radula of *P. stellatus*. Scale bars 50 μm (**A**); 10 μm (**B–D**).

**Figure 8.**
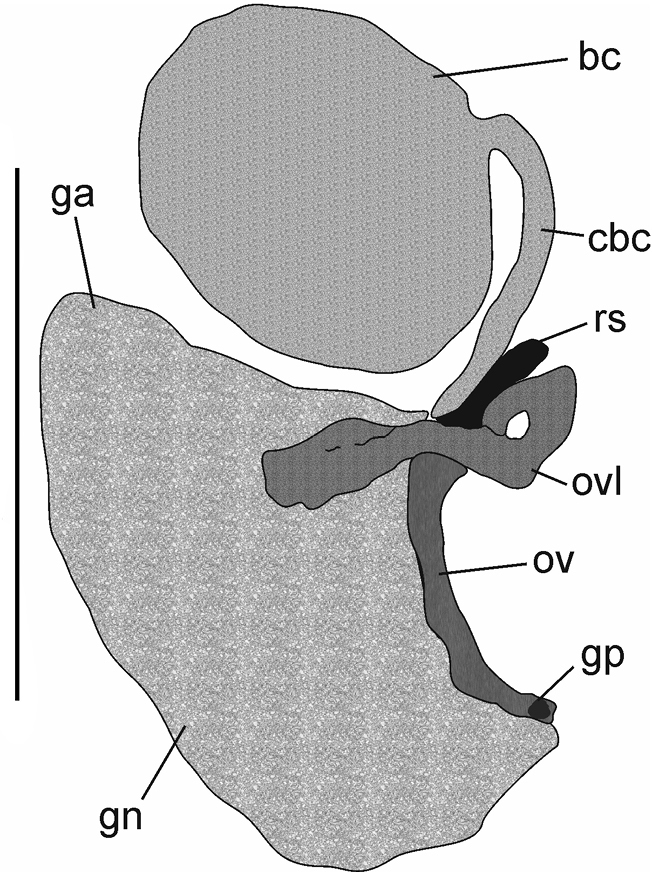
Renal and pallial section of the female reproductive organs of *Paladilhiopsis stellatus X*. Abbreviations: bc – bursa copulatrix, cbc – duct of bursa, ga – albuminoid gland, gn – nidamental gland, gp – gonoporus, ov – oviduct, ovl – loop of (renal) oviduct, rs – seminal receptacle (in the position of Radoman’s (1983) rs_1_ – distal seminal receptacle). Scale bar: 500 μm.

**Figure 9.**
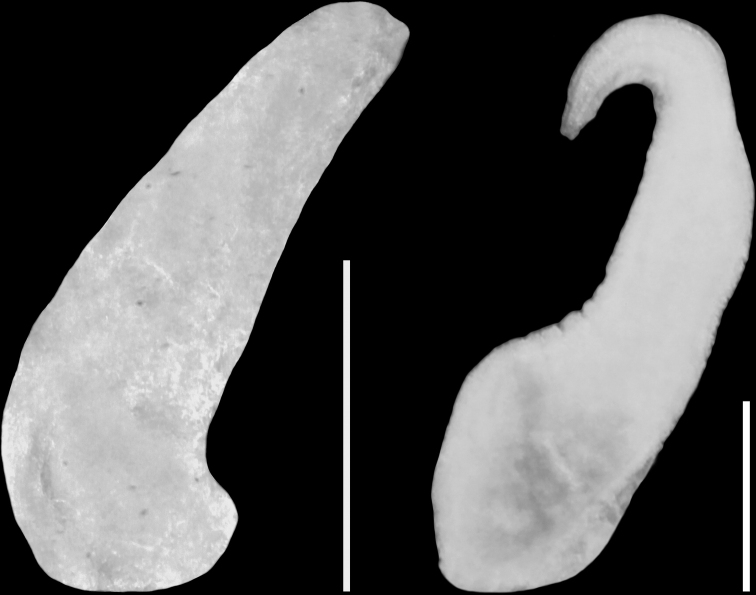
Penes of *Paladilhiopsis stellatus*. Scale bars: 200 μm.

Figures 6A–P , 7B–D , 8 , 9

###### Note.

mOTU A (Fig. 5); localities 1 and 2; Fig. 6A–P; GenBank numbers: COI: MW741724–MW741738; H3: MW776417–MW776423

###### Type locality.

Spring Zvezda, above left bank of Cetina River, Slime, Omiš district, Croatia (43°26'13"N, 16°44'26"E) (Fig. 2B, locality 2).

***Holotype*.** Ethanol-fixed specimen (Fig. 6C), interstitially in the gravel below the bottom of the spring; J. Grego, A. Falniowski, R. Ozimec, M. Olšavský, J. Olšavská leg.; 08 August 2020; NHMW113632.

***Paratypes*.** Type locality; J. Grego, A. Falniowski, R. Ozimec, M. Olšavský, J. Olšavská leg.; 08 August 2020; ten ethanol-fixed paratypes in the collection of the Department of Malacology of Jagiellonian University, dry specimens: ZMUJ-M.2633-2642, HNHM/2 specimens, NHMW-MO 113627/2 specimens, SMF362990/2 specimens, PG/2 specimens, JG-F1628 /158 specimens.

###### Other material.

Studena spring, concrete well ca. 80 m from road at hillside, Slime, Omiš district Croatia; 43°25'43"N, 16°51'59.74"E; J. Grego, A. Falniowski, R. Ozimec, M. Olšavský, J. Olšavská leg.; 08 August 2020; JG F1626/20 specimens (Fig. 2A, locality 1); Studena spring, stony catchment, left bank of Cetina River, Slime, Omiš district Croatia; 43°25'45.48"N, 16°51'59.57"E; J. Grego, A. Falniowski, R. Ozimec, M. Olšavský, J. Olšavská leg.; 08 August 2020; JG-F1621/3 fragmented specimens (ca. 80 m uphill from locality 1).

###### Diagnosis.

Shell minute, elongate-conic (turriform), distinguishable from the geographically close *Paladilhiopsis elongata* (Kuščer, 1933) from spring Jadro near Split (HR) by less sinuated lateral labral profile, more inflated and more prominent body whorls, and a more elongate pyramidal shape. From *P. solida* Kuščer, 1933 from Vrelo Buna in Blagaj (BiH) differs by its smaller less conical shell with less prominent body whorl and slightly sinuate lateral labral margin adapically preceding. Can be distinguished from *P. pretneri* Bole & Velkovrh, 1987 from Antunovići near Kozice, Makarska district (HR) by its longer, more elongate shell and proportionally smaller body whorl. The receptaculum seminis long and tubular in shape, similar to that of *P. bosniaca* (Clessin, 1910), and different from the bulbous one with a long duct as in *P. grobeni* Kuščer, 1928.

###### Description.

***Shell*** (Fig. 6A–P) up to 2.94 mm high and 1.02 mm broad, ovate-conic (turriform), white or whitish, translucent, thin-walled, consisted of ca. six whorls, growing slowly and regularly, and separated by moderately deep but sharply marked suture. Spire high and conic, apex narrow, body whorl height less than 0.5 of the shell height. Aperture small, prosocline, oval, or elongated oval in shape, peristome complete and thin, umbilicus slit-like. Shell surface smooth, glossy, with growth lines hardly visible.

***Measurements*** of holotype and sequenced and illustrated shells presented in Table 4. Shell variability slight, marked mostly in breadth: height proportion of the shell and the aperture (Fig. 6A–P).

***Radula*** (Fig. 7B–D) taenioglossate, typical of *Paladilhiopsis*, with numerous, long, and cusps. Rhachis formula:

$\frac{\left(5)4-1-4(5\right)}{1-1}$ or $\frac{4-1-4}{1-1}$

Basal cusps widely triangular and massive, median cusp at the cutting edge 2 × longer than the adjacent ones, lateral tooth formula: 3 – 1 – 4, the largest cusp prominent, nearly 2 × longer than the adjacent ones, on the inner marginal tooth ca. 18 large cusps similar to the ones on the rhachis, ca. 20 smaller and more slender cusps on the outer marginal tooth.

***Soft parts morphology and anatomy*.** Body white, with no pigment, with no eyes. Female reproductive organs (Fig. 8) typical of the genus *Paladilhiopsis* (Hofman et al. 2018), with unpigmented, long, and narrow renal oviduct, large bursa copulatrix, although less elongated than in *P. grobbeni*, and with its duct (characteristically for *Paladilhiopsis*) lying proximally, single small distal receptaculum seminis (in the position of rs_1_ after Radoman 1973), with the outlet to the oviduct close to the outlet of the duct of the bursa copulatrix, and shortened accessory gland complex. Long narrow loop of the renal oviduct and small short seminal receptacle are characteristic of the species. The simple penis (Fig. 9) typical of the genus, without any outgrowth, tapering, in the form of an elongated triangle.

###### Derivatio nominis.

The specific epithet *stellatus* refers to *stella*, the Latin word for star for the name of the type locality, Zvezda Spring, *zvezda* which means star in Croatian.

###### Known distribution.

Besides the type locality (locality 2: Spring Zvezda, above left bank of Cetina, Croatia), found also at locality 1: Studena spring, left bank of Cetina River, Slime, Croatia, 43°25'45.48"N, 16°51'59.57"E.

###### Remarks.

Molecularly this mOTU is the sister clade of the mOTU B (*Paladilhiopsis gittenbergeri*), but genetic distance is high, 11.3% for COI 3.5% for H3.

##### Paladilhiopsis arion

Taxon classificationAnimaliaLittorinimorphaMoitessieriidae

Rysiewska et Osikowski sp. nov.

7822FBE4-057F-5AB1-B6A0-77AC092C87CA

http://zoobank.org/7901fa54-583b-4825-b482-d82413291808

Figures 6Q–S , 7A

###### Note.

mOTU C (Fig. 5); locality 3; Fig. 6Q-S; GenBank numbers: COI: MW741739– MW741740; H3: MW776424–MW776425

###### Type locality.

Vrelo “Lušac” (Gučina), BiH (42°42'04"N, 18°21'27"E) (locality 3).

***Holotype*.** Ethanol-fixed specimen (Fig. 6Q), interstitially (pumped with Bou-Rouch pump), in the gravel below the bottom of the spring; 10 Sept 2019; A. Falniowski, A. Rysiewska and A. Osikowski leg., voucher number: ZMUJ-M.2643.

***Paratypes*.** Five empty shells, in the collection of the Department of Malacology of Jagiellonian University, voucher numbers: ZMUJ-M.2644-2648.

###### Diagnosis.

Shell minute, elongate-conic (turriform), with relatively narrow spire, whose breadth grows rapidly, acute narrow apex, and narrow but long aperture with prominent lip, distinguishable from the geographically close *Paladilhiopsis matejkoi* Grego & Glöer, 2019 from Nemila Spring, Herceg Novi (MNE) and *P. montenegrinus* (Schütt, 1959, described as *Saxurinator*) from Bileća (BiH) by its more conical shell shape with slightly pagoda-shaped whorls, sharper apex, and deeper suture. From *P. matejkoi* it can additionally be differentiated by its more declined aperture.

###### Description.

***Shell*** (Fig. 6Q–S) 2.51 mm high and 0.91 mm broad, elongate-conic (turriform), white, translucent, thin-walled, consisted of ca. six and half whorls, growing slowly and regularly in their height, but growing rapidly in breadth, separated by moderately deep but sharply marked suture. Spire high and slim, apex acute, body whorl height ca. 0.38 of the shell height. Aperture narrow but long, peristome complete, forming a prominent lip, umbilicus slit-like. Shell surface smooth, glossy, with clearly visible broad growth lines, forming ribs (Fig. 7A).

***Measurements*** of holotype and sequenced and illustrated shells provided in Table 4.

**Table 4.** Shell measurements of the sequenced *Paladilhiopsis*, and the holotypes (in bold). Measurements as shown in Fig. 6: a – shell height, b – body whorl breadth, c – aperture height, d – spire height, e – aperture breadth, α – apex angle measured between the lines tangential to the spire, β – angle between the body whorl suture and the line perpendicular to the columella.

	a	b	c	d	e	α	β
*Paladilhiopsis stellatus*							
**holotype**	**2.32**	**0.83**	**0.71**	**1.18**	**0.53**	**91**	**18**
2H58	2.94	1.02	0.92	1.57	0.73	85	22
2H59	2.33	0.95	0.79	1.09	0.70	88	18
2H60	2.18	0.84	0.63	1.13	0.45	95	18
2H61	2.38	0.9	0.74	1.24	0.61	90	20
2i19	1.99	0.79	0.61	1.03	0.46	92	19
2i21	2.50	0.89	0.74	1.32	0.60	95	20
2i22	2.41	0.91	0.69	1.27	0.63	96	18
2i23	2.41	0.83	0.71	1.32	0.55	96	23
2i24	2.34	0.88	0.72	1.21	0.61	93	18
2i25	2.19	0.83	0.71	1.12	0.57	91	18
2i26	2.54	0.92	0.78	1.36	0.59	96	21
2i27	2.11	0.79	0.69	1.07	0.53	88	18
2i28	2.35	0.83	0.71	1.34	0.48	89	19
M	2.36	0.87	0.73	1.23	0.57	91.79	19.29
SD	0.225	0.065	0.074	0.145	0.083	3.534	1.684
Min	1.99	0.79	0.61	1.03	0.45	85	18
Max	2.94	1.02	0.92	1.57	0.73	96	23
*Paladilhiopsis arion*							
**holotype**	**2.28**	**0.88**	**0.75**	**1.23**	**0.69**	**84**	**21**
2G19	2.28	0.85	0.78	1.32	0.74	76	18
2G22	2.51	0.91	0.74	1.49	0.71	90	19

***Soft parts morphology and anatomy*.** The body is white, without pigment, with no eyes. The arrangement of pellets in the rectum characteristic for the Moitessieriidae (Boeters and Gittenberger 1990). The anatomy is unknown.

###### Derivatio nominis.

The specific epithet *arion* refers to the ancient name of River Trebišnjica, which in classical antiquity was known as the *Arion*, rising and sinking through its course before resurfacing at various places from the Neretva to the coast; Trebišnjica is adjacent to the type locality.

###### Known distribution.

Only the type locality.

###### Remarks.

Molecularly this mOTU is the sister clade of the mOTU D (*Paladilhiopsis matejkoi*), with genetic distances 5.9% for COI 1.6% for H3.

## Discussion

Molecularly, the strict monomorphism in the mitochondrial locus in the mOTU A is striking: the same haplotype occurs at two localities. The monomorphism in the stygobiont organism is often recorded, but usually only a single or few specimens are available, thus one cannot deny the possibility that the picture is biased by too many samples. In our materials as many as 13 species from locality 2 (and two from locality 1) were identical, confirming the real lack of genetic polymorphism. The presented photographs show the shell morphology in one population, which is also slightly variable.

As demonstrated by Falniowski (1987, 1990, 2018) the details of the reproductive organs, such as the shape of the receptaculum and/or bursa copulatrix, are hardly useful in species-level taxonomy of the Truncatelloidea: they are either uniform above species level or variable within a species. This is clearly visible in the moitessieriid gastropods, whose anatomy is even more simplified, due to miniaturisation, than in most of the truncatelloidean families. The female reproductive organs of *Paladilhiopsis stellatus* are practically identical as in *P. bosniaca*, and only slightly different from that of *P. grobbeni*.

Progress in molecular taxonomy methods has led to the development of many tools for species delimitation. Among the most widely used, the automatic barcode gap discovery (ABGD), Poisson tree processes (PTP), as well as the general mixed Yule coalescent (GMYC) were proposed (Pons et al. 2006). All of them have many limitations, like sensitivity to gene flow and the ratio of the population size to the divergence time (PTP, GMYC), tendency to under- or over-split species (ABGD, GMYC), and many others (e.g., Pentinsaari et al. 2017; Luo et al. 2018). We have not been able to use the GMYC methods because we have no strictly ultrametric input tree. For this reason, we have used the alternative PTP technique and, for comparison, also the ABGD delimitation. Low numbers of DNA fragments also possibly weakened our results. On the other hand, increasing the number of loci as well as the sample size per species results only in a modest benefit for species delimitation methods (Luo et al. 2018). This, together with identical results obtained with two methods based on different assumptions, supports the reliability of our inference of species distinction. This is also enhanced by comparisons of the genetic distances. They are quite high, especially for *P. stellatus*. The p-distance between different *Paladhiliopsis* species varied from 0.013 to 0.125 (Hofman et al. 2018), and our values are higher than or close to the mean. Moreover, for other snails closely related to the Moitessieriidae, the estimated threshold p-distances for species delimitation applying COI were strikingly low: ca. 0.015 in the genus *Bythinella* (Bichain et al. 2007) and 0.023 (Hurt 2004) for *Pyrgulopsis*. All the above, coupled with the differences in morphology, supports the descriptions of these two new species.

The Truncatelloidea are one of the great examples of the Balkan biodiversity (Bănărescu 2004). New species from this region, including the ones whose distinctiveness is supported by molecular data, are still being described from this area (e.g., Hofman et al. 2019, 2020; Falniowski et al. 2021). The Mediterranean Basin Hotspot is one of the 34 biodiversity hotspots in the world (Nyers et al. 2000. Many factors are responsible for the high level of the biological diversity in this region, e.g., location at the intersection of two major landmasses (Eurasia and Africa), a complex geological history, huge topographical diversity and altitudinal differences, unique climate with cool, wet winters and hot, dry summers. Unfortunately, human activity has drastically affected aquatic habitats decreasing this biodiversity (e.g., Szarowska and Falniowski 2011). Rivers and their floodplains, lakes, wetlands, and especially springs, exploited as sources of water and contaminated, offer increasingly bad conditions for the aquatic fauna. In this context, studies of this diminishing biodiversity are important and urgent.

## Supplementary Material

XML Treatment for Paladilhiopsis stellatus

XML Treatment for Paladilhiopsis arion
